# Biodegradation of Iprodione and Chlorpyrifos Using an Immobilized Bacterial Consortium in a Packed-Bed Bioreactor

**DOI:** 10.3390/microorganisms11010220

**Published:** 2023-01-15

**Authors:** Marcela Levío-Raimán, Cristian Bornhardt, M. Cristina Diez

**Affiliations:** 1 Biotechnological Research Center Applied to the Environment (CIBAMA-BIOREN), Universidad de La Frontera, Temuco 4780000, Chile; 2 Chemical Engineering Department, Universidad de La Frontera, Temuco 4780000, Chile

**Keywords:** pesticides, biodegradation, chlorpyrifos, iprodione, immobilized bacterial consortium, packed-bed bioreactor

## Abstract

This work provides the basis for implementing a continuous treatment system using a bacterial consortium for wastewater containing a pesticide mixture of iprodione (IPR) and chlorpyrifos (CHL). Two bacterial strains (*Achromobacter spanius* C1 and *Pseudomonas rhodesiae* C4) isolated from the biomixture of a biopurification system were able to efficiently remove pesticides IPR and CHL at different concentrations (10 to 100 mg L^−1^) from the liquid medium as individual strains and free consortium. The half-life time (*T*_1/2_) for IPR and CHL was determined for individual strains and a free bacterial consortium. However, when the free bacterial consortium was used, a lower *T*_1/2_ was obtained, especially for CHL. Based on these results, an immobilized bacterial consortium was formulated with each bacterial strain encapsulated individually in alginate beads. Then, different inoculum concentrations (5, 10, and 15% *w/v*) of the immobilized consortium were evaluated in batch experiments for IPR and CHL removal. The inoculum concentration of 15% *w/v* demonstrated the highest pesticide removal. Using this inoculum concentration, the packed-bed bioreactor with an immobilized bacterial consortium was operated in continuous mode at different flow rates (30, 60, and 90 mL h^−1^) at a pesticide concentration of 50 mg L^−1^ each. The performance in the bioreactor demonstrated that it is possible to efficiently remove a pesticide mixture of IPR and CHL in a continuous system. The metabolites 3,5-dichloroaniline (3,5-DCA) and 3,5,6-trichloro-2-pyridinol (TCP) were produced, and a slight accumulation of TCP was observed. The bioreactor was influenced by TCP accumulation but was able to recover performance quickly. Finally, after 60 days of operation, the removal efficiency was 96% for IPR and 82% for CHL. The findings of this study demonstrate that it is possible to remove IPR and CHL from pesticide-containing wastewater in a continuous system.

## 1. Introduction

Pesticides are widely used in agricultural activities to reduce pests and diseases in crops [1]. However, the excessive use and inadequate handling of pesticides have negative effects on the environment and human health. Residues of pesticides and their transformation products can enter the human body and ecosystem, where they can bioaccumulate [2]. Some fruit/vegetable processing industries also generate pesticide-containing wastewater due to the high volume of clean water used to eliminate dust and residual pesticides in the products. If this wastewater does not receive adequate treatment prior to its disposal, the pesticides contained within can contaminate surface water and groundwater [3]. Pesticide-containing wastewater is considered difficult to treat due to its complex chemical composition, poor biodegradability, and high concentration of organic compounds [4]. The removal of these contaminants is of great concern worldwide due to the impacts of such contaminants on natural resources and human health. According to Zheng et al. [4], due to the high organic matter content and high toxicity of pesticide-containing wastewater, the most efficient removal method must consider combined treatments. Therefore, a combination of physical (adsorption, extraction, electro-dialysis, etc.) and chemical (Fenton method, electrochemical advanced oxidation method, etc.) pretreatments alongside biological (aerobic, anaerobic, among others) treatments are the most promising methods to remove and decrease the quantity of pesticides in wastewater [5]. However, the high energy consumption and high-cost operation of physical and chemical treatments makes the implementation of these methods unattainable in many agroindustries. To provide a solution to this problem, new and affordable technologies are needed to clean and prevent water resource contamination. In this context, the biopurification system (BPS) plays a significant role in avoiding point-source contamination by pesticides [6,7,8].

The BPS is based on the adsorption and degradation capacity of an organic biomixture generally composed of soil, peat, and wheat straw in respective volumetric proportions of 1:1:2. This organic biomixture has demonstrated high efficiency in pesticide removal under different conditions (e.g., high pesticide concentrations, repeated pesticide applications, and pesticide mixtures) [8]. The pesticide degradation in BPS is performed by microorganisms, mainly bacteria and fungi strains, that proliferate in the organic biomixture [9,10] and are recognized as pesticide degraders [11,12,13,14]. The microorganism capacity for pesticide degradation is determined by its adaptation to contaminated environments, microbial diversity, and enzyme production [15]. The use of bacterial strains in biodegradation processes has increased in recent years due to the ability of bacteria to degrade pesticides such as chlorpyrifos (CHL), diazinon, and iprodione (IPR), among many others [16,17,18]. Additionally, in some cases, the formulation of microbial consortia is considered a viable alternative to pesticide degradation because consortia are more productive and robust than single strains [19,20,21]. Recent studies reported that microbial consortia and single strains isolated from a BPS can efficiently remove pesticides such as atrazine, carbofuran, and glyphosate with the highest degradation (>90%) using mixed consortia with the strains *Ochrobactrum* sp. DGG-1-3, *Ochrobactrum* sp. Ge-14, *Ochrobactrum* sp. B18, and *Pseudomonas citronellolis* strain ADA-23B [22]. Therefore, the microorganisms isolated from a BPS were found to tolerate pesticides and could be used as an inoculum to increase efficiency in pesticide treatment.

In addition to determining suitable microorganisms for the degradation of pesticides, it is necessary to determine the best technological process to remove these contaminants from wastewater. The use of bioreactors for pesticide degradation has been studied to avoid and reduce the presence of pesticides in wastewater effluent. The packed-bed reactor (PBR) is considered to be an adequate model system for pesticide degradation because it favors the retention of higher microorganism concentrations in the reactor [23,24,25,26]. However, the capacity of bacterial strains isolated from a BPS to formulate a microbial consortium for use in pesticide treatment in a continuous system remains almost unstudied. Additionally, the implementation of an efficient and cost-effective method for the treatment of pesticide-containing wastewater is essential to avoid negative impacts.

Consequently, the aim of this study was to evaluate the use of *Achromobacter spanius* C1 and *Pseudomonas rhodesiae* C4 strains isolated from a BPS as an immobilized bacterial consortium for pesticide-containing wastewater treatment to improve IPR and CHL degradation and reduce their half-life time. In this study, two compounds were used as models of degradation: IPR (fungicide) and CHL (insecticide). The bacterial strains were evaluated as individual strains, free bacterial consortium, and immobilized bacterial consortium to improve IPR and CHL degradation and reduce their half-life time. Then, we used a packed-bed bioreactor loaded with the bacterial consortium immobilized in alginate beads to evaluate and verify the performance and extent of the biotransformation of pesticides in a continuous system. The results of this study will reveal the technical considerations for designing a pesticide-containing wastewater treatment system.

## 2. Materials and Methods

### 2.1. Pesticides

Analytical grade (99%) commercial standards of IPR, 3,5-dichloroaniline (3,5-DCA), CHL, and 3,5,6-trichloro-2-pyridinol (TCP) were purchased from Sigma-Aldrich (St. Louis, MO, USA) for high-performance liquid chromatography (HPLC) analysis. Stock solutions of 1000 mg L^−1^ were dissolved in acetone 100% and sterilized by filtration through a 0.22 µm polytetrafluoroethylene (PTFE) filter. For biodegradation assays, commercial products of IPR (Rovral 50 WP) and CHL (Troya 4EC) were acquired from Agan Chemicals Manufacturers Ltd. (Ashdod, Israel) (Table 1). Stock solutions of commercial products at 10,000 mg L^−1^ dissolved in methanol were filtered through a 0.22 µm PTFE filter and stored at 4 °C until use. All solvents used for HPLC analysis were purchased from Merck-Sigma (St. Louis, MO, USA).

### 2.2. Bacterial Strains and Culture Media

Bacterial strains *Achromobacter spanius* strain C1 (GenBank accession number: MK110041) and *Pseudomonas rhodesiae* strain C4 (GenBank accession number: MK110043), previously identified by the 16S rRNA gene [14], were used in this study. These bacterial strains were previously isolated from BPS used during the last three years for the treatment of commercial IPR and CHL (50 mg kg^−1^) [27] and were maintained at 4 °C on plate count agar (PCA) containing (per liter) 5 g tryptone, 2.5 g yeast extract, 1 g glucose, and 15 g agar adjusted at pH 7.0. Luria Bertani medium (LB) containing (per liter) 5.0 g NaCl, 5.0 g yeast extract, and 10.0 g casein peptone at pH 7 was used as the culture medium for all evaluations with the individual strains, with the free and immobilized bacterial consortium using a liquid medium.

### 2.3. Pesticide Degradation by Individual Bacterial Strains and Free Bacterial Consortium

Pesticide degradation was evaluated using each bacterial strain individually (C1 and C4) and with the free bacterial consortium. Using plate count agar (PCA), a fresh bacterial inoculum was cultivated in an LB medium on an orbital shaker at 130 rpm and 28 °C for 24 h in darkness [14]. Assays were carried out in 100 mL flasks with 50 mL of LB medium supplemented with a pesticide mixture of IPR and CHL in increasing concentrations of 10, 20, 50, and 100 mg L^−1^ each. Each flask (in triplicate) was inoculated with 1% *v/v* of bacteria individually or in a free bacterial consortium, as appropriate. The flasks were incubated for 96 h at 28 °C on an orbital shaker at 100 rpm in darkness. Samples (5 mL) were taken at different times of incubation to analyze the pesticide concentrations and metabolites via HPLC. For pesticide degradation, the kinetic parameters were calculated. 

### 2.4. Immobilization of Bacterial Strains

For strain immobilization, fresh inocula of the *A. spanius* C1 and *P. rhodesiae* C4 strains were inoculated in 100 mL of LB medium at 1% *v/v* and incubated at 28 °C for 48 h with constant agitation at 130 rpm. Then, each bacterial strain was centrifuged at 10,000 rpm for 10 min. Each resulting microorganism pellet containing a biomass concentration of 2 g L^−1^ was washed 3 times with sterile distilled water and resuspended in 20 mL of sterile distilled water. Each bacterial strain was mixed with a sodium alginate solution (3% *v*/*v*) for immobilization in alginate beads. Then, each mixture with the corresponding bacterial strain was homogenized using a magnetic stirrer and transferred dropwise into 0.1 M CaCl_2_, forming beads immediately (3–4 mm in diameter). Each bacterial strain was immobilized individually. The beads were maintained in CaCl_2_ solution under agitation for 6 h. Then, the beads were washed 3 times and stored in a 0.9% physiological solution in closed and sterile containers at 4 °C. Beads without microorganisms were used as the control. The shape and surface structure of the encapsulated strains were analyzed via scanning electron microscopy (SEM VP-SEM SU 3500, Hitachi-Tokyo, Japan). For subsequent analyses, the immobilized bacterial consortium was formulated using each bacterial strain immobilized individually in the same ratio of 1:1.

### 2.5. Pesticide Degradation by the Immobilized Bacterial Consortium in Batch Mode

The assay with the immobilized bacterial consortium formulated with each bacterial strain immobilized individually was conducted in 100 mL flasks containing 50 mL of LB medium (in triplicate). A pesticide mixture of IPR and CHL was added to the flasks at a concentration of 50 mg L^−1^ each. Then, inoculum concentrations of 5, 10, and 15% *w/v* of immobilized beads of *A. spanius* C1 and *P. rhodesiae* C4 strains were added into each flask in a ratio of 1:1 for pesticide degradation assays. Beads without microorganisms were used as a control. The incubation was conducted for 120 h in the dark at 28 ± 1 °C on an orbital shaker at 100 rpm. At different times, samples (2 mL) were collected to quantify pesticides and their metabolites using HPLC.

From this assay, the inoculum concentration selected was 15% *w/v* of immobilized beads of each *A. spanius* C1 and *P. rhodesiae* C4 strains in a ratio of 1:1, for subsequent assays.

### 2.6. Pesticide Degradation by Immobilized Bacterial Consortium in Continuous Mode

The experimental setup for the continuous bioreactor was performed as described by Levio-Raiman et al. [27]. Borosilicate glass columns (5 cm internal diameter, 15 cm length, working volume of 295 mL) were packed with the immobilized bacterial consortium. To pack the bioreactor, we considered an inoculum concentration (selected in Section 2.5) of 15% *w/v* for each immobilized strain (separately) of *A. spanius* C1 and *P. rhodesiae* C4 in an equal ratio of 1:1. The immobilized consortium was packed homogeneously in the bioreactor, and the fluid was passed through and between the beads in down-flow mode. Additionally, glass wool was placed at the top and bottom of the columns to prevent preferential flow and loss of the packed immobilized bacterial consortium. Additionally, the bioreactor was sealed with parafilm to prevent evaporation. Aeration and mixing were not supplied to the packed-bed bioreactor. The pesticide solution was passed continuously through the columns in the down-flow mode using a peristaltic pump (Brand Biobase Model FPP3, YZ1515X) at increasing flow rates of 30, 60, and 90 mL h^−1^. The flow rate was changed every 20 days. The bioreactor operation was carried out for 60 days at room temperature (approximately 22 °C) without a heating system. Samples (2 mL) were collected in the influent and effluent to quantify the pesticides and their metabolites via HPLC. The criterion to change the flow rate was observed based on the bioreactor behavior; when a steady condition was reached with a concentration relatively constant at the effluent, the flow rate was changed. Performance was evaluated in terms of removal efficiency according to Equation (1):

(1) $$RE\;\left(\%\right)=\left(\frac{C_{i}-C_{e}}{C_{i}}\right)\cdot 100\;$$

where *C_i_* and *C_e_* are, respectively, the inlet and outlet concentrations of the pesticides in the bioreactor.

### 2.7. Analyses of Pesticides and Metabolites

Samples were taken, treated, and analyzed according to the method described by Briceño et al. [14]. The analysis was conducted using an HPLC Merck Hitachi L-2130 equipped with a Rheodyne 7725 injector and a Merck Hitachi L-2455 diode array detector. Separation was achieved using a C18 column (Chromolit RP-8e, 4.6 μm × 100 mm). The mobile phase was 70% 1 mM ammonium acetate and 30% acetonitrile injected at a flow rate of 1 mL min^−1^. The column temperature was maintained at 30 ± 1 °C, and the detector was set for data acquisition at 290 nm for IPR and CHL. Instrument calibrations and quantifications were performed against pure reference standards (0.01–10 mg L^−1^) for each pesticide. Average recovery amounts for the pesticides were as follows: IPR, 92 ± 2.2%; and CHL, 101 ± 0.7%. The limit of quantification (LOQ) was determined using the smallest concentration of the analyte in the test sample, which induced a signal that was ten times higher than the background noise level (IPR = 0.238 mg L^−1^ and CHL = 0.214 mg L^−1^). The limit of detection (LOD) was 0.089 mg L^−1^ for IPR and 0.081 mg L^−1^ for CHL.

### 2.8. Kinetics and Statistical Analysis

The removal of IPR and CHL from the liquid medium was fitted to the first-order kinetic model according to Equation (2):

(2) $$\ln\frac{C_{t}}{C_{0}}=e^{-kt}\;$$

where *C*_0_ is the initial concentration of the pesticides in the liquid medium, *Ct* is the concentration of the pesticides at time *t*, *k* is the degradation rate constant (h^−1^), and *t* is the reaction time (h). The degradation half-life (*T*_1/2_) is the time in which the pesticide concentrations in the liquid medium are reduced by 50% and was calculated using Equation (3).

(3) $$T_{1/2}=\frac{\;\ln2\;}{k}\;$$

Data were statistically analyzed using analysis of variance (ANOVA), and three replicates were compared using Tukey’s minimum significant differences test (*p* ≤ 0.05). Statistical analyses were performed using SPSS statistical software version 17.

## 3. Results

### 3.1. Pesticide Removal by Individual Bacterial Strains and Free Bacterial Consortium

**Figure 1:**
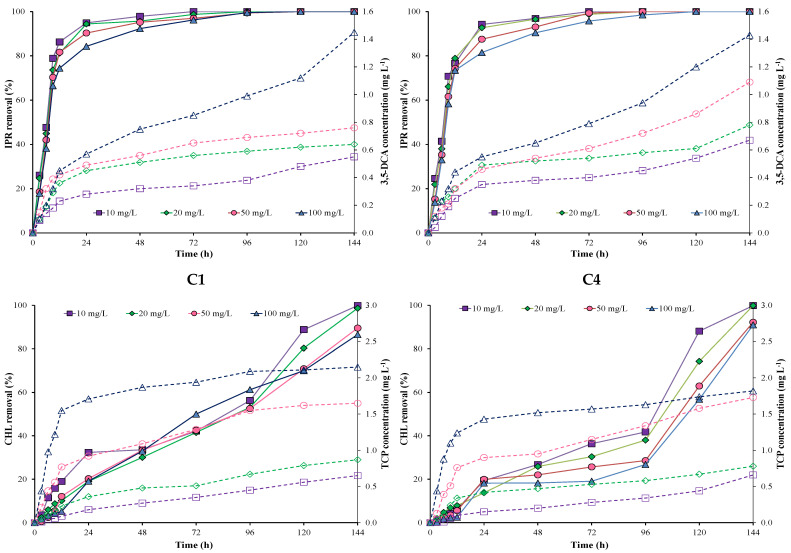
Pesticide removal (symbol filled with a continuous line) and metabolite production (empty symbol with a dotted line) when using individual *A. spanius* **C1** (**left**) and *P*. *rhodesiae* **C4** (**right**) strains in a liquid medium. Iprodione (IPR) and chlorpyrifos (CHL) were added (in mixture) at concentrations of 10, 20, 50, and 100 mg L^−1^ each.

Figure 1 shows IPR and CHL removal and metabolite production by each individual *A. spanius* C1 and *P. rhodesiae* C4 strains at different incubation times and pesticide concentrations. The results showed that IPR removal was higher and faster than CHL removal during the first hours with both individual strains (C1 and C4). IPR removal was >80% in the first 24 h and reached 100% after 96 h in all evaluated concentrations for both individual strains.

Conversely, CHL removal was initially slow for both individual strains (C1 and C4) in all concentrations evaluated. In general, CHL removal using the C1 strain was better and faster than that using the C4 strain. Additionally, the increase in CHL concentrations (10, 20, 50, and 100 mg L^−1^) influenced CHL removal during the first 24 h for both strains. CHL removal was 100% at 10 and 20 mg L^−1^ of CHL for both strains. However, CHL removal decreased at 50 and 100 mg L^−1^ of CHL for both strains. For example, CHL removal for the C1 strain reached 89.5 and 86.5% at 50 and 100 mg L^−1^ of CHL, respectively. For the C4 strain, CHL removal reached 92.2 and 90.9% at 50 and 100 mg L^−1^ of CHL, respectively.

The metabolites 3,5-DCA and TCP were measured for all evaluated concentrations (Figure 1). The values obtained for the 3,5-DCA concentration fluctuated between 0.54 and 1.43 mg L^−1^ in both strains. Additionally, in both strains, we observed a higher increase in the 3,5-DCA concentration at 100 mg L^−1^ of IPR. On the other hand, TCP concentrations fluctuated between 0.59 and 2.14 mg L^−1^ for the C1 strain and 0.52 and 1.75 mg L^−1^ for the C4 strain. Additionally, in both strains, we observed a higher increase in TCP concentration at 50 and 100 mg L^−1^ of CHL.

**Figure 2:**
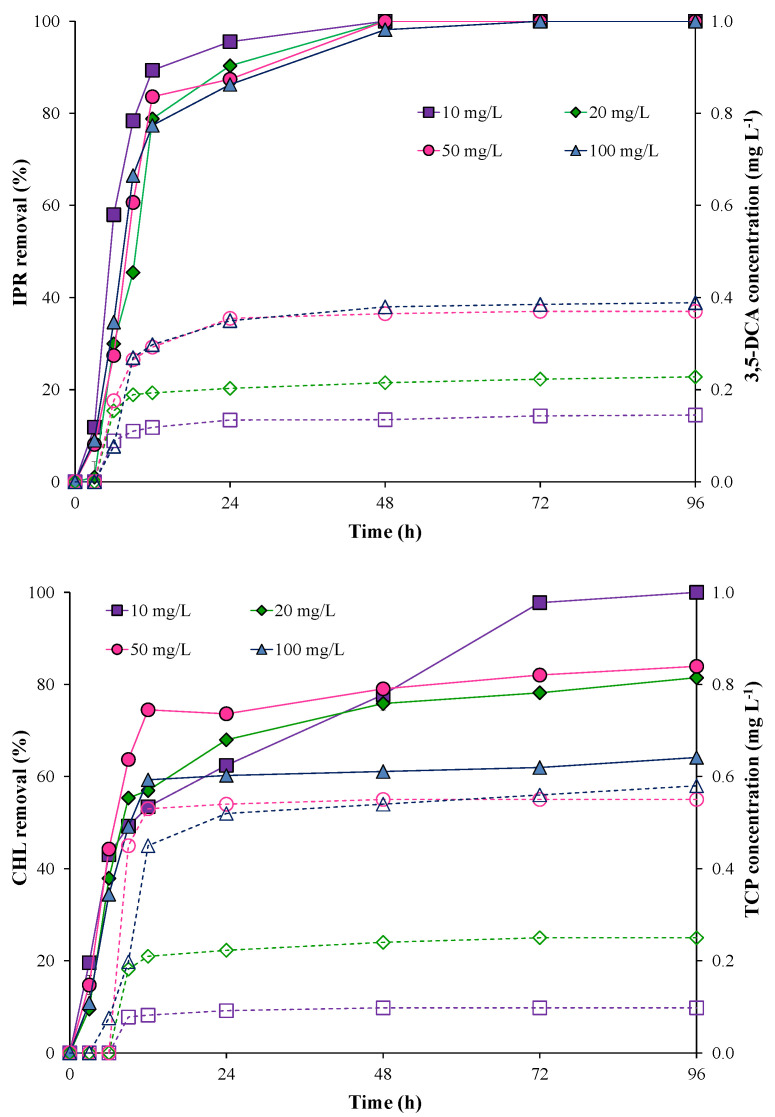
Pesticide removal (symbol filled with a continuous line) and metabolite production (empty symbol with a dotted line) when using the free bacterial consortium in a liquid medium. Iprodione (IPR) and chlorpyrifos (CHL) were added (in mixture) at initial concentrations of 10, 20, 50, and 100 mg L^−1^ each.

The pesticide removal and metabolite production using the free bacterial consortium are shown in Figure 2. The results showed that IPR removal with the free bacterial consortium was fast and high (>70%) during the first 12 h and independent of IPR concentration. Additionally, after 72 h, IPR removal was 100% for all IPR concentrations evaluated.

Conversely, CHL removal was slow and minimal (<50%) during the first 12 h for all concentrations evaluated with the free bacterial consortium. Overall, increases in CHL concentrations negatively influenced CHL removal. Indeed, at the end of the assay, CHL removal was 100% only for 10 mg L^−1^ of CHL. For 20, 50, and 100 mg L^−1^ CHL concentrations, removal was 81.44, 83.93, and 64.14%, respectively.

In terms of metabolite production, after 24 h, the maximum 3,5-DCA concentration was 0.38 and 0.39 mg L^−1^ at 50 and 100 mg L^−1^ of IPR, respectively. The metabolite TCP showed similar behavior, with maximum values of 0.55 and 0.58 mg L^−1^ at 50 and 100 mg L^−1^ of CHL, respectively. After 24 h, the 3,5-DCA and TCP metabolite concentrations were observed to be stable.

Table 2 shows the first-order kinetics parameters, *k* and *T*_1/2_, calculated for IPR and CHL using the individual bacterial strains and the free bacterial consortium. IPR removal by the C1 strain was characterized by *k* (h^−1^) values between 0.10 and 0.16 h^−1^ and *T*_1/2_ (h) values between 4.29 and 7.11 h. On the other hand, IPR removal by the C4 strain was characterized by *k* (h^−1^) values between 0.07 and 0.12 h^−1^ and *T*_1/2_ (h) values between 8.49 and 12.93 h. For the free bacterial consortium, IPR removal showed *k* (h^−1^) values between 0.20 and 0.29 h^−1^ and *T*_1/2_ (h) values between 8.63 and 8.81 h. Significant differences (*p* < 0.05) were observed only for *T*_1/2_ at 50 mg L^−1^ of IPR concentration for the individual C1 and C4 strains and 100 mg L^−1^ for the free bacterial consortium.

On the other hand, CHL removal by strain C1 (Table 2) was characterized by a *k* with values between 0.01 and 0.02 h^−1^. Additionally, the *T*_1/2_ fluctuated between 109.92 and 231.12 h. On the other hand, CHL removal by bacterial strain C4 was characterized by a *k* that fluctuated between 0.01 and 0.02 h^−1^ and *T*_1/2_ with values between 145.68 and 277.44 h. Generally, the behavior of the kinetic parameters (*k* and *T*_1/2_) for CHL removal were similar between the individual strains (C1 and C4) evaluated. However, the *T*_1/2_ decreased significantly (*p* < 0.05), with the bacterial consortium being lower than individual strains. CHL removal by the bacterial consortium was characterized by a *k* that fluctuated between 0.16 and 0.20 h^−1^ and a *T*_1/2_ between 10.08 and 12.96 h. Therefore, with the free bacterial consortium, the *T*_1/2_ of CHL decreased more than ten times compared to that of individual strains. These results confirm that the free bacterial consortium significantly accelerated CHL removal. In this context, the ability to remove CHL was evaluated as follows: free bacterial consortium > strain C1 > strain C4.

### 3.2. Pesticide Removal by Immobilized Bacterial Consortium

**Figure 3:**
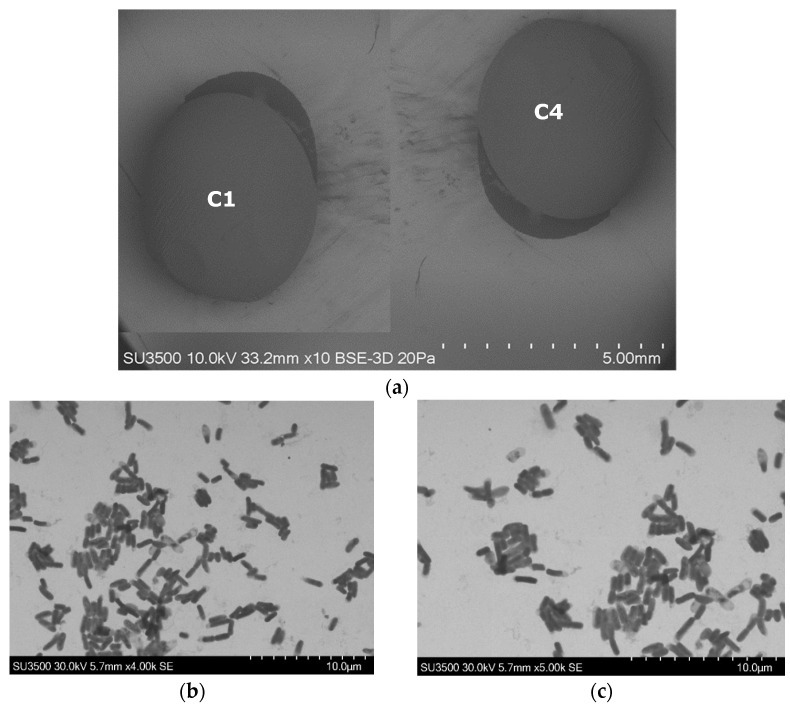
Electron scan micrographs of alginate beads with bacterial strains *A. spanius* C1 and *P. rhodesiae* C4 immobilized individually; (**a**) morphological surfaces of alginate beads (strains C1 and C4); (**b**) inside view of alginate bead with strain C1; (**c**) inside view of alginate bead with strain C4.

IPR and CHL removal was evaluated with an immobilized bacterial consortium formulated with each bacterial strain (C1 and C4) immobilized individually. Figure 3 shows an SEM image with the external and internal view of the alginate beads with each bacterial strain immobilized. Here, the alginate beads present a spherical form (Figure 3a), and the inside of the beads indicate the proliferation and growth of each strain immobilized: the C1 strain (Figure 3b) and C4 strain (Figure 3c).

**Figure 4:**
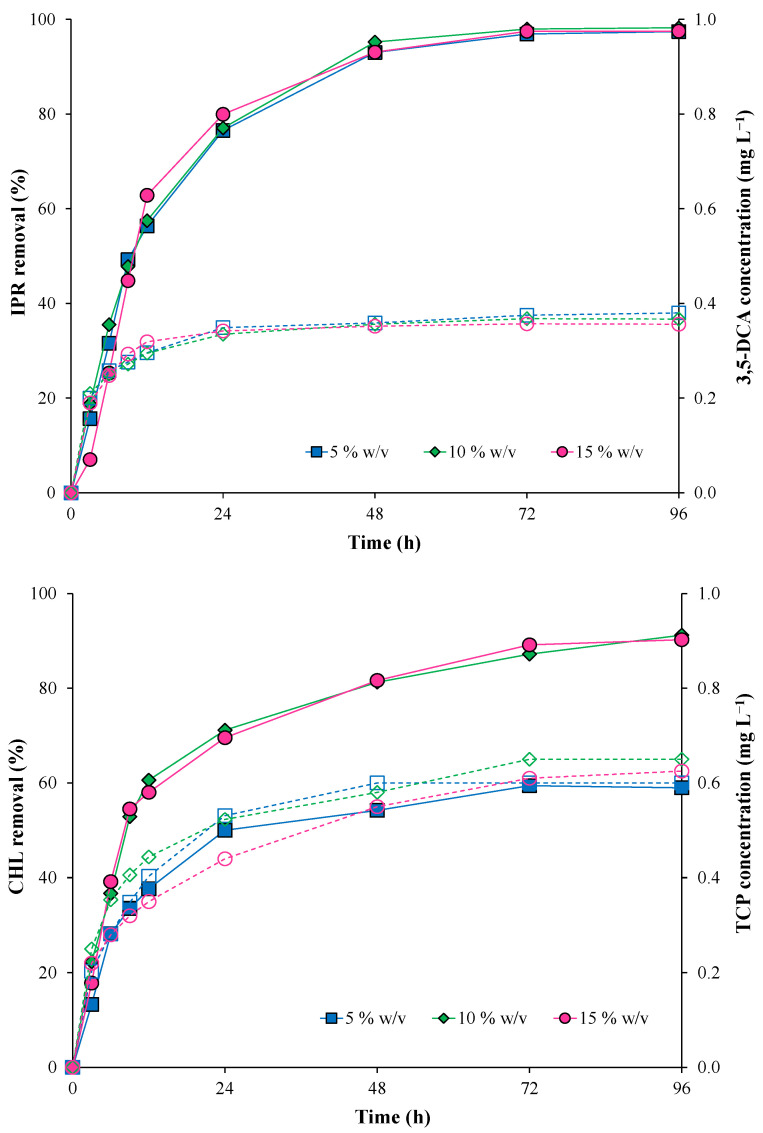
Pesticide removal (symbol filled with continuous line) and metabolite production (empty symbol with a dotted line) with the immobilized bacterial consortium at different inoculum concentrations (5, 10, and 15% *w/v*). Iprodione (IPR) and chlorpyrifos (CHL) were added (in mixture) at an initial concentration of 50 mg L^−1^ each.

Figure 4 shows the results obtained for IPR and CHL removal, evaluating different inoculum concentrations of the immobilized bacterial consortium (5, 10, and 15% *w/v*) at 50 mg L^−1^ (each pesticide). IPR removal did not present significant differences when the inoculum concentration increased. At the end of the assay, IPR was removed entirely (Figure 4). IPR removal was characterized by *T*_1/2_ values of 11.8, 11.5, and 10.8 h for 5, 10, and 15% *w/v* inoculum concentration, respectively (Table 3).

Conversely, CHL removal was influenced by an increase in the inoculum concentration. CHL removal was higher at inoculum concentrations of 10 and 15% *w/v*. Additionally, the *T*_1/2_ for CHL was reduced from 24.42 h with 5% *w/v* to 9.29 and 9.10 h with 10 and 15% *w/v*, respectively. 

In addition, 3,5-DCA and TCP metabolites were detected during all evaluated times. For the 3,5-DCA metabolite, the concentration remained stable from 24 h with a maximum value of 0.38 mg L^−1^ at the end of the assay. For the TCP metabolite, the maximum concentrations were obtained at the end of the assay with values of 0.60, 0.65, and 0.63 mg L^−1^ for 5, 10, and 15% *w/v* inoculum concentrations, respectively.

In summary, the lowest *T*_1/2_ for both pesticides was observed under the highest inoculum concentration (15% *w/v*) (Table 3); therefore, this inoculum concentration was used in the subsequent assay.

### 3.3. Pesticide Degradation via the Immobilized Bacterial Consortium in a Packed-Bed Bioreactor

**Figure 5:**
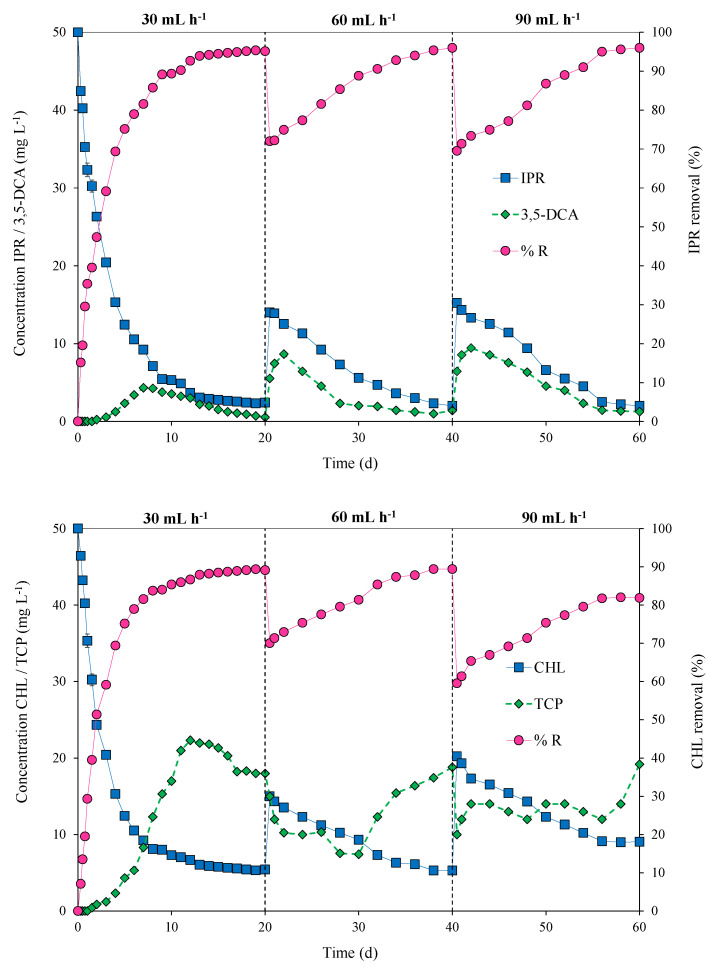
Pesticide concentrations/metabolites (mg L^−1^) in the effluent and removal efficiency (% R) for iprodione (IPR) and chlorpyrifos (CHL) fed (in mixture) at a concentration of 50 mg L^−1^ each in a packed-bed bioreactor operated at different flow rates (30, 60, and 90 mL h^−1^) over time (0 to 60 days) and inoculated with an immobilized bacterial consortium at an inoculum concentration of 15% *w/v*.

The packed-bed bioreactor was operated in continuous mode at flow rates of 30, 60, and 90 mL h^−1^ for the pesticide mixture of IPR and CHL at a concentration of 50 mg L^−1^ each. Figure 5 shows the variation in pesticide concentrations, removal efficiency (%), and metabolite concentrations at different flow rates over time (0 to 60 days).

During the first 20 days, the bioreactor was operated at 30 mL h^−1^ to facilitate microbial growth and provide steady-state conditions. In this context, a steady state was achieved for IPR on Day 12 and for CHL on Day 13, reaching a pesticide removal rate of 95% for IPR and 89% for CHL. On Day 20, the flow rate was duplicated, and decreased removal for both pesticides was observed. However, a quick recovery was observed starting from Days 34 and 38 for IPR and CHL, respectively. On Day 40, the flow rate again increased to 90 mL h^−1^, and a dip in removal efficiency was observed. In the case of IPR, the efficiency recovery was fast and reached 96% removal on Day 60. Conversely, for CHL, the recovery was slow and only reached 82% removal.

The production of metabolites 3,5-DCA and TCP during bioreactor operation was also quantified. In the case of 3,5-DCA, on Day 7, we observed a peak concentration that reached 4.32 mg L^−1^ in the first 20 d of operation at a flow rate of 30 mL h^−1^. Then, peaks in 3,5-DCA concentration were observed after the increased flow rate. For a flow rate of 60 mL h^−1^, the peak concentration for 3,5-DCA was 8.65 mg L^−1^ on Day 22. Additionally, for a flow rate of 90 mL h^−1^, the peak concentration of 3,5-DCA was 9.43 mg L^−1^ at 42 d of operation. For TCP, the concentration presented several variations and was not observed as a steady state. Indeed, a slight accumulation of TCP was observed. TCP reached concentrations of 22.32, 18,01, and 19.21 mg L^−1^ at flow rates of 30, 60, and 90 mL h^−1^, respectively (Figure 5).

## 4. Discussion

Biodegradation is generally considered cheap, environmentally friendly, and easy to implement compared to other methods, both physical and chemical [28,29]. The microbial strains involved in pesticide biodegradation are primarily fungi, bacteria, and actinomycetes [15,30]. However, some authors indicated that bacteria are more effective in responding to xenobiotics in their immediate environments and can either tolerate/evade high concentrations of xenobiotics or remove them through biodegradation [15,29,31]. Several batch studies have reported the biodegradation of some pesticides such as CHL, 2,4-dichlorophenoxyacetic (2,4-D), malathion, and IPR using microbial *Pseudomonas nitroreducens*, *Cupriavidus necator, Bacillus,* and *Achromobacter* strains, respectively, isolated from contaminated sites [28,29,32,33]. However, few studies have evaluated pesticide degradation in a continuous system using different bioreactor configurations to treat pesticide-containing wastewater.

In this study, two bacterial strains were isolated from an organic biomixture of BPS used for pesticide treatment over several years. This organic biomixture contains many active microorganisms and represents an attractive approach because these bacteria have already adapted to interact with high pesticide concentrations [8,22,27]. Both bacterial strains used in this study, *A. spanius* C1 and *P. rhodesiae* C4, were able to remove high pesticide concentrations of IPR and CHL, and this activity was found to be more effective when both strains were immobilized and used as a consortium. The organic biomixture used in the BPS is a good source of microorganisms adapted to pesticides, as reported in other studies [3,22,34]. Additionally, Briceño et al. [14] reported the same bacterial strains used in this study, *Achromobacter spanius* C1 and *Pseudomonas rhodesiae* C4, as promising microorganisms for the biodegradation of IPR and CHL.

On the other hand, recent studies reported that microbial consortia and individual bacterial strains isolated from a BPS can efficiently remove pesticides such as atrazine, carbofuran, and glyphosate with high degradation (>90%) results using a mixed consortium with the strains *Ochrobactrum* sp. DGG-1-3, *Ochrobactrum* sp. Ge-14, *Ochrobactrum* sp. B18, and *Pseudomonas citronellolis* strain ADA-23B [22]. In this study, similar behavior was observed, as the individual strains *A. spanius* C1 and *P. rhodesiae* C4 and free bacterial consortium were able to efficiently remove both pesticides. However, with the bacterial consortium, we observed a lower half-life for CHL and the highest efficiency of removal for both pesticides. In addition, with individual bacterial strains and the free bacterial consortium, we observed the presence of the main degradation metabolites (3,5-DCA and TCP) of the pesticides studied. The presence of these metabolites confirmed that the bacterial consortium was able to biodegrade IPR and CHL and also their metabolites 3,5-DCA and TCP, which reduce their concentration over time. In fact, 3,5-DCA was degraded completely, and only in TCP was a slight increase and accumulation in the liquid medium observed.

IPR degradation using strains from the genera *Pseudomonas* and *Achromobacter* has been poorly studied and was only reported by Mercadier et al. [35] and Campos et al. [18,33], respectively. Mercadier et al. [35] evaluated the degradation pathway of IPR by bacterial strains consisting of *Pseudomonas fluorescens*, *Pseudomonas* sp., and *Pseudomonas paucimobilis*. The authors showed that IPR was microbially hydrolyzed to 3,5-DCA through the formation of three intermediate metabolites, isopropylamine and 3,5-dichlorophenylcarboxiamide (Metabolite I), which were initially produced. The latter was subsequently transformed into 3,5-dichlorophenylurea acetate (Metabolite II), which was ultimately hydrolyzed to 3,5-DCA. A similar pathway was confirmed by Campos et al. [33] when evaluating *Arthrobacter* strain C1 and *Achromobacter* strain C2. Campos et al. [33] indicated that *Arthrobacter* sp. strain C1 is a key IPR degrader able to obtain complete degradation within 8 and 24 h assays. This strain maintained its degradation capacity under a wide range of temperatures and pH values. Additionally, the *Achromobacter* sp. strain C2 was able to co-metabolize IPR in a rich medium after 240 h, ultimately allowing transformation to 3,5-DCA. In our study, the *Achromobacter spanius* strain C1 presented faster IPR degradation and a lower *T*_1/2_ (between 4.29 and 7.11 h) than the *Pseudomonas rhodesiae* C4 strain. Moreover, the metabolite 3,5-DCA was detected during all evaluated times for individual strains, which is consistent with what was described by Campos et al. [33] for the same genus of bacteria. On the other hand, the free bacterial consortium was faster in IPR degradation, with *k* values between 0.20 and 0.28 h^−1^. In general, the degradation process with the consortium was carried out faster due to each strain sharing biochemical steps to mineralize toxic contaminants through enzyme interactions [36]. Yang et al. [37] further reported that IPR was degraded through a pathway via a novel amidase enzyme present in some bacteria such as *Paenarthrobacter* sp. strain YJN-5. In agreement with our results, the isolated bacterial *A. spanius* C1 and *P. rhodesiae* C4 strains adapted to IPR could constitute a source of hydrolytic enzymes (e.g., esterase and phosphatase) [14] responsible for IPR transformation. However, we did not investigate which enzymes may be involved in IPR degradation. A future assay could elucidate the biochemical mechanisms of biodegradation.

In terms of CHL removal by *A. spanius* C1 and *P. rhodesiae* C4, both strains demonstrated strong pesticide degradation abilities. Indeed, some authors have reported the same microbial genera for CHL degradation [31,38,39,40]. Akbar and Sultan [38] reported that the *Achromobacter xylosoxidans* strain JCp4 isolated from pesticide-contaminated agricultural fields was able to degrade 84.4% CHL from an initial concentration of 100 mg L^−1^ in 10 days. Moreover, Aswathi et al. [31] reported 97% removal of CHL by a *Pseudomonas nitroreducens* AR-3 strain isolated from pesticide-contaminated agricultural soil. Additionally, Rayu et al. [39] reported the biodegradation of CHL and TCP with the same genera *Pseudomonas* sp. The authors reported that the *Pseudomonas* sp. 4H1-M3 strain presented high CHL degradation without another carbon or nitrogen source. Therefore, there is evidence indicating the effective use of these genera of bacterial strains in CHL and TCP degradation. Additionally, *Pseudomonas* sp. has been commonly described as a CHL degrader [40].

According to Briceño et al. [14], the *Achromobacter* sp. C1 strain evidenced high CHL removal, which could be associated with the presence and activity of the enzyme alkaline phosphatase, as this enzyme is a phosphomonoesterase that regulates CHL degradation through the hydrolysis of O–P bonds [41,42]. Similarly, the presence of diverse enzymes in *Pseudomonas* sp. (strain C4) could influence fast degradation and, therefore, reduce the *T*_1/2_ required for pesticide reduction [14]. Nonetheless, despite that these microbial genera can degrade IPR and CHL efficiently as individual strains, our results suggest that as a microbial consortium, the efficiency increases significantly (*p* < 0.05).

The obtained results show a significant reduction in the *T*_1/2_ of CHL using a free and immobilized consortium with respect to individual strains. Some authors argued that this difference could be attributed to the cooperative metabolism of a microbial consortium that is considered more beneficial due to the possible combination of different enzymes produced by individual strains able to degrade the toxic compound [43,44]. Additionally, the lower *T*_1/2_ could be explained by the microbial ability to divide their functions and distribute more complex metabolic tasks using carbon sources simultaneously, thus improving degradation efficiency compared to individual strains [43,45].

The immobilization of microorganisms on various supports as bio-polymeric beads has shown potential to improve biodegradation efficiency in terms of sustainability compared to free cells. Additionally, immobilization enhances cell viability and increases cell tolerance to higher concentrations of pollutants [46]. In this respect, Ca-alginate beads have been extensively studied for their efficient immobilization of microbial cells due to their low toxicity, ease of use, and low cost [22,27]. Ca-alginate beads act like a slow-release delivery system, where the bacterial cells or their enzymes are slowly released into the medium, enhancing the rate of degradation, tolerance to higher pesticide concentrations, and biomass reusability/recuperation [47].

Under these considerations, in our study, we performed the immobilization of bacterial strains individually for each strain, due to their different specific growth rates (µ max), and to ensure that each bacterial strain would have one equal biomass concentration and a homogeneous distribution inside the alginate bead. In this way, we promoted a high density of cells in the support [48,49]. Additionally, we considered the specific growth rates of 0.15 and 0.29 h^−1^ for the C1 and C4 strains, respectively, where the C4 strain grows faster than the C1 strain and, consequently, can achieve a higher biomass concentration over time. Therefore, we immobilized these strains separately to avoid unequal growth inside the alginate bead.

On the other hand, cell immobilization enables the operation of bioreactors at flow rates that are independent of the microorganisms, thus enhancing tolerance against higher concentrations of toxic compounds compared to free cells [47]. Therefore, to operate the packed bioreactor, we used the immobilized bacterial consortium at an inoculum concentration of 15% *w/v*.

The performance of the PBR in pesticide-containing wastewater treatment depends on many factors, such as flow rate, pesticide concentration, packing material, and bed dimensions [50]. Additionally, the PBR’s operation is simple, offers a high yield, and can be easily scaled up from a laboratory-scale procedure [26]. In this study, the performance of a PBR in continuous mode was evaluated based on the removal efficiency of IPR and CHL at a 50 mg L^−1^ concentration each (in mixture) and at different inlet flow rates (30, 60, and 90 mL h^−1^) over a period of 60 days. The bacterial consortium immobilized in alginate beads at an inoculum concentration of 15% (*w/v*) was effective in the treatment of pesticide-containing wastewater contaminated with a mixture of IPR and CHL. The biodegradation of CHL by bacteria in a PBR was studied previously by Yadav et al. [51] who investigated the biodegradation of CHL via *Pseudomonas* sp. in both batch and continuous modes using bioreactors packed with polyurethane foam pieces. The authors found that the bioreactor was sensitive to flow rate fluctuations but able to recover its performance quickly. However, TCP accumulation affected the bioreactor performance. We obtained similar results showing unstable behavior in terms of TCP concentration with a tendency to accumulate. The initially higher TCP accumulation levels during the first few days of the experiment could be attributed to the slow acclimation of bacterial strains to TCP [52]. However, after the initial acclimation, the bacterial strains also started degrading TCP because a decreasing concentration was observed. Huang et al. [53] reported that TCP has antibacterial properties and an inhibitory effect on microbial communities. Therefore, the gradual accumulation of TCP could inhibit bacterial activity and, consequently, affect bioreactor performance due to the high toxicity, persistence, and water solubility of TCP [51,54]. Such toxic effects of TCP could be attributed to the release of chlorine atoms from TCP during the degradation process adversely affecting the growth rates of microorganisms, which have used TCP as a source of energy [41]. Nonetheless, the growth rates were observed to quickly recover, which indicates that the selected strains offered high degradation of the initial compound and its metabolites.

According to the available literature, this study is the first report evaluating IPR degradation via an immobilized bacterial consortium in a bioreactor. The results obtained indicated a great affinity between the IPR and bacterial strains selected. Indeed, the IPR removal was independent of flow rate fluctuations, and 3,5-DCA decreased and did not accumulate. This behavior demonstrated that it is possible to efficiently remove IPR and 3,5-DCA from pesticide-containing wastewater.

## 5. Conclusions

According to our results, microorganisms that are tolerant and able to degrade pesticides exist in the biopurification system. The bacterial *Achromobacter spanius* C1 and *Pseudomonas rhodesiae* C4 strains were observed to efficiently remove IPR and CHL. Additionally, the formulated bacterial consortium improved pesticide degradation and decreased the half-life time of both pesticides. The evaluation of a packed-bed bioreactor with an immobilized bacterial consortium demonstrated that it is possible to efficiently remove a pesticide mixture of IPR and CHL in a continuous system. However, an improvement in the TCP degradation process is required to guarantee complete metabolite removal that does not affect water quality and the environment.

Despite this, this study represents an effective and interesting approach to the treatment of pesticide-containing wastewater.

## Figures and Tables

**Table 1:** Chemical properties of pesticides used in this study.

Characteristics	Iprodione	Chlorpyrifos
Chemical class	Dicarboximide	Organophosphate
Molecular formula	C_9_H_11_C_l3_NO_3_PS	C_13_H_13_C_l2_N_3_O_3_
Chemical structure	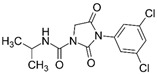	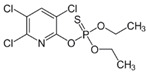
Water solubility (mg L^−1^)	6.80	1.05
Molecular weight (g mol^−1^)	330	351
*T*_1/2_ (d) in soils	36	50
*T*_1/2_ (d) in biomixture *	15.5	19.8
GUS	2.7	0.17
Kow (Log P)	3.0	4.7
Koc	700	8100

Solubility in water at 20 °C; *T*_1/2_: half-life time, GUS: Groundwater Ubiquity Score, leaching potential index; K_oc_: Adsorption coefficient; K_ow:_ n-octanol/water partition coefficient. Source: Pesticide Properties DataBase (PPDB). * Obtained from Diez et al. [8].

**Table 2:** First-order kinetics parameters for iprodione (IPR) and chlorpyrifos (CHL) removal at different concentrations of 10, 20, 50, and 100 mg L^−1^ with the individual bacterial strains (C1 and C4) and free bacterial consortium.

		C1			C4			Free Bacterial Consortium		
Pesticide	Concentration (mg L^−1^)	*k* (h^−1^)	*T*_1/2_ (h)	R^2^	*k* (h^−1^)	*T*_1/2_ (h)	R^2^	*k* (h^−1^)	*T*_1/2_ (h)	R^2^
IPR	10	0.11 ± 0.01	6.27 ± 0.10 **a**	0.998	0.12 ± 0.03	12.40 ± 0.02 **a**	0.985	0.29 ± 0.00	8.81 ± 0.11 **a**	0.960
	20	0.11 ± 0.01	6.40 ± 0.20 **a**	0.978	0.11± 0.02	12.93 ± 0.01 **a**	0.994	0.28 ± 0.01	8.74 ± 0.21 **a**	0.997
	50	0.16 ± 0.02	4.29 ± 0.20 **b**	0.999	0.09 ± 0.01	8.49 ± 0.05 **b**	0.995	0.25 ± 0.02	8.63 ± 0.01 **a**	0.965
	100	0.10 ± 0.01	7.11 ± 0.10 **a**	0.987	0.07 ± 0.01	12.77 ± 0.02 **a**	0.982	0.20 ± 0.00	8.71 ± 0.02 **a**	0.991
CHL	10	0.01 ± 0.001	112.56 ± 2.40 **a**	0.981	0.01 ± 0.001	145.68 ± 2.64 **a**	0.992	0.17 ± 0.01	10.56 ± 1.88 **a**	0.975
	20	0.01 ± 0.001	109.92 ± 2.88 **a**	0.983	0.02 ± 0.001	170.16 ± 5.52 **a**	0.990	0.20 ± 0.01	10.08 ± 1.42 **a**	0.991
	50	0.02 ± 0.001	198.24 ± 3.60 **b**	0.994	0.01 ± 0.001	259.42 ± 2.88 **b**	0.998	0.16 ± 0.01	12.96 ± 1.79 **a**	0.962
	100	0.01 ± 0.001	231.12 ± 2.64 **b**	0.998	0.01 ± 0.001	277.44 ± 2.40 **b**	0.991	0.17 ± 0.01	12.24 ± 1.98 **a**	0.955

The ± represents the standard deviation of the means of three replicates. The values with different letters indicate significant differences (*p* < 0.05, Tuckey test) considering individual strains and the free bacterial consortium; *T*_1/2_: half-life time, *k*: degradation rate constant.

**Table 3:** First-order kinetics parameters for iprodione (IPR) and chlorpyrifos (CHL) removal at a concentration of 50 mg L^−1^ each, inoculated with the immobilized bacterial consortium at different inoculum concentrations (5, 10, and 15% *w/v*).

	IPR			CHL		
Inoculum Concentration (% *w/v*)	*k* (h^−1^)	*T*_1/2_ (h)	R^2^	*k* (h^−1^)	*T*_1/2_ (h)	R^2^
5	0.14 ± 0.02	11.78 ± 0.01 **a**	0.997	0.09 ± 0.000	24.42 ± 0.02 **a**	0.996
10	0.15 ± 0.01	11.51 ± 0.02 **a**	0.998	0.10 ± 0.001	9.29 ± 0.10 **b**	0.991
15	0.16 ± 0.03	10.78 ± 0.01 **a**	0.995	0.12 ± 0.001	9.10 ± 0.05 **b**	0.998

The ± represents the standard deviation of the means of three replicates. The values with different letters indicate significant differences (*p* < 0.05, Tuckey test) based on inoculum concentration. *T*_1/2_: half-life time, *k*: degradation rate constant.

## Data Availability

Not applicable.
